# Rare Syringyl Acylated Flavonol Glycosides from the Aerial Parts of *Leonurus japonicus* Houtt

**DOI:** 10.3390/molecules18032967

**Published:** 2013-03-04

**Authors:** Yi Zhang, Shen Deng, Lu Qu, Ya-Ting An, Chun-Hua Wu, Li-Feng Han, Xiu-Mei Gao, Tao Wang

**Affiliations:** 1 Tianjin State Key Laboratory of Modern Chinese Medicine, 312 Anshanxi Road, Nankai District, Tianjin 300193, China; 2 Tianjin Key Laboratory of TCM Chemistry and Analysis, Institute of Traditional Chinese Medicine, Tianjin University of Traditional Chinese Medicine, 312 Anshan Road, Nankai District, Tianjin 300193, China

**Keywords:** *Leonurus japonicus*, syringyl acylated flavonol glycosides, TG accumulation inhibitory effects, HepG2 cells

## Abstract

Five new syringyl acylated flavonol glycosides, named leonurusoides A (**1**), B (**2**), C (**3**), D (**4**), and E (**5**), together with one known one **6** were obtained from the aerial parts of *Leonurus japonicus*. Their structures were elucidated by chemical and spectroscopic methods (UV, IR, HRESI-TOF-MS, 1D and 2D NMR). Compounds **1**−**6** showed triglyceride (TG) accumulation inhibitory effects in free fatty acid-induced HepG2 cells.

## 1. Introduction

**Figure 1:**
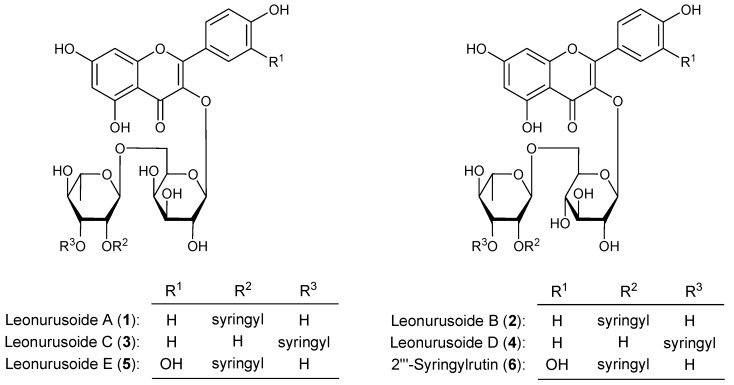
Chemical structures of **1**−**6**.

*Leonurus japonicus* Houtt is from the Lamiaceae family. As a Traditional Chinese Medicine (TCM), it has been commonly used to treat disorders of mammary gland [1]. Several metabolites such as alkaloids [2,3,4,5,6], flavonoids [6,7,8,9], and other compounds [10] have been isolated from this plant. During the course of our characterization studies on the bioactive constituents from the aerial parts of *L. japonicus*, five new syringyl acylated flavonol glycosides, named leonurusoides A (**1**), B (**2**), C (**3**), D (**4**), and E (**5**), together with the known one, 2'''-syringylrutin (**6**) (Figure 1) were obtained. This paper deals with the isolation and TG accumulation inhibitory effects in HepG2 cells elucidation of these compounds.

## 2. Results and Discussion

The aerial parts of *L. japonicus* were finely cut and refluxed with 50% ethanol-water. Evaporation of the solvent under reduced pressure provided a 50% ethanol-water extract, which was partitioned into a CHCl_3_-H_2_O (1:1, v/v) mixture to furnish a CHCl_3_-soluble fraction and an aqueous phase. The aqueous soluble fraction was subjected to column chromatography (CC) over macroporous resin D101, normal- and reversed-phase CC, and finally HPLC to give five new compounds, leonurusoides A (**1**), B (**2**), C (**3**), D (**4**), and E (**5**), together with the known one, 2'''-syringylrutin (**6**).

*Leonurusoide A* (**1**) was isolated as a yellow powder with negative optical rotation [[α]^25^_D_ –13.0° (*c* = 0.54, MeOH)]. Its molecular formula was determined to be C_36_H_38_O_19_ by positive-ion HRESI-TOF-MS (*m/z* 775.2076 [M+H]^+^, calcd for C_36_H_39_O_19_ 775.2080). The IR spectrum exhibited absorption bands typical of OH (3356 cm^−1^), ester carboxyl group (1699 cm^–1^), *α*,*β*-unsaturated ketone (1654 cm^–1^), aromatic ring (1608, 1516, 1457 cm^–1^), and an *O*-glycosidic linkage (1056 cm^–1^). The UV data of **1** showed the characteristic absorption maxima of a kampferol aglycon at 351 nm (4.00) and 268 nm (4.24). Acid hydrolysis of **1** with 1 M HCl afforded D-galactose and L-rhamnose, whose absolute configurations were determined by HPLC analysis using an optical rotation detector [11,12]. The ^1^H- (500 MHz) and ^13^C-NMR (125 MHz) spectra of **1** (DMSO-*d*_6_, Table 1), which were assigned by various NMR experiments including ^1^H-^1^H COSY, HSQC, and HMBC spectra, indicated there was a kampferol part [*δ* 6.14 (1H, br. s, H-6), 6.34 (1H, br. s, H-8), 6.81 (2H, d, *J* = 8.5 Hz, H-3',5'), 8.02 (2H, d, *J* = 8.5 Hz, H-2',6'), 12.53 (1H, br. s, 5-OH)], together with an *α*-L-rhamnopyranosyl and a *β*-D-galactopyranosyl moieties [*δ* 4.57 (1H, br. s, H-1'''), 5.30 (1H, d, *J* = 7.5 Hz, H-1'')] in the structure. On the other hand, the appearance of a methoxyl signal at *δ* 3.81 (6H, s, 3'''',5''''-OC*H*_3_) and a sharp aromatic singlet signal at *δ* 7.19 (2H, s, H-2'''',6'''') in the ^1^H-NMR spectrum, together with the signals at *δ* 55.9 (3'''',5''''-O*C*H_3_), 107.0 (C-2'''',6''''), 119.2 (C-1''''), 140.9 (C-4''''), 147.4 (C-3'''',5''''), 164.7 (C-7'''') in the ^13^C-NMR one, indicated the presence of a syringyl group. Furthermore, the assignment of glycoside protons was determined by proton-proton correlations observed from ^1^H-^1^H COSY and HSQC experiments. Finally, the linkages of *α*-L-rhamnopyranosyl, *β*-D-galactopyranosyl, and syringyl moieties were clarified on the basis of HMBC experiment, which showed long-range correlations between *δ*_H_ 5.30 (H-1'') and *δ*_C_ 133.2 (C-3); *δ*_H_ 4.57 (H-1''') and *δ*_C_ 65.3 (C-6''), and *δ*_H_ 4.92 (1H, br. d, *ca*. *J* = 3 Hz, H-2''') and *δ*_C_ 164.7 (C-7''''). On the basis of above mentioned, the structure of leonurusoide A was determined as kampferol-3-*O*-(2'''-syringyl)-*α*-L-rhamnopyranosyl(1→6)-*β*-D-galactopyranoside (**1**).

**Table 1:** ^1^H and ^13^C-NMR data for compounds **1** and **2** in DMSO-*d*_6_ (500 MHz for ^1^H and 125 MHz for ^13^C).

No.	1		2	
	*δ*_C_	*δ*_H_ (*J* in Hz)	*δ*_C_	*δ*_H_ (*J* in Hz)
2	156.2	—	156.3	—
3	133.2	—	133.1	—
4	177.0	—	177.1	—
5	161.0	—	161.0	—
6	99.1	6.14 (1H, br. s)	98.9	6.16 (1H, br. s)
7	165.9	—	165.1	—
8	93.9	6.34 (1H, br. s)	93.8	6.34 (1H, br. s)
9	156.5	—	156.5	—
10	103.2	—	103.5	—
1'	120.8	—	120.8	—
2',6'	130.7	8.02 (2H, d, 8.5)	130.7	7.96 (2H, d, 8.5)
3',5'	114.9	6.81 (2H, d, 8.5)	114.9	6.83 (2H, d, 8.5)
4'	159.8	—	159.8	—
1''	102.2	5.30 (1H, d, 7.5)	101.5	5.32 (1H, d, 7.5)
2''	71.0	3.57 (1H, dd, 7.5, 9.0)	74.1	3.18 (1H, dd, 7.5, 9.0)
3''	72.9	3.43 (1H, dd, 3.0, 9.0)	76.3	3.23 (1H, dd, 9.0, 9.0)
4''	67.9	3.65 (1H, m, overlapped)	69.7	3.08 (1H, dd, 9.0, 9.0)
5''	73.2	3.63 (1H, m, overlapped)	75.6	3.29 (1H, m)
6''	65.3	3.32 (1H, dd, 5.0, 9.0)	66.8	3.38 (1H, dd, 5.0, 9.0)
		3.66 (1H, m, overlapped)		3.73 (1H, br. d, *ca*. 11)
5-OH		12.53 (1H, br. s)		12.54 (1H, br. s)
1'''	96.9	4.57 (1H, br. s)	97.6	4.53 (1H, br. s)
2'''	72.5	4.92 (1H, br. d, *ca*. 3)	72.5	4.95 (1H, br. d, *ca*. 3)
3'''	68.4	3.63 (1H, m, overlapped)	68.6	3.61 (1H, dd, 3.0, 9.0)
4'''	72.4	3.26 (1H, dd, 9.5, 9.5)	72.4	3.24 (1H, dd, 9.0, 9.5)
5'''	68.6	3.52 (1H, m)	68.4	3.41 (1H, m)
6'''	18.0	1.15 (3H, d, 6.0)	17.8	1.07 (3H, d, 6.0)
1''''	119.2	—	119.4	—
2'''',6''''	107.0	7.19 (2H, s)	107.1	7.19 (2H, s)
3'''',5''''	147.4	—	147.4	—
4''''	140.9	—	140.7	—
7''''	164.7	—	164.7	—
-OCH_3_	55.9	3.81 (6H, s)	56.0	3.82 (6H, s)

**Figure 2:**
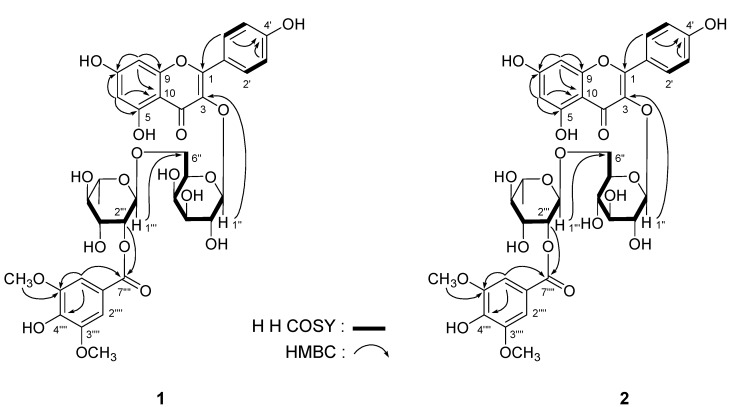
The main ^1^H-^1^H COSY and HMBC correlations of compounds **1** and **2**.

*Leonurusoide B* (**2**) was obtained as a yellow powder and exhibited a negative rotation [[α]^25^_D_ –16.6° (*c* = 0.21, MeOH)]. The IR spectrum of **2** indicated the presences of carboxyl (1699 cm^–1^), *γ*-pyrone (1653 and 1569 cm^–1^) functions, and a glycoside structure (3218, 1055 cm^–1^). HRESI-TOF-MS analysis suggested that **2** had the same molecular formula as **1** [C_36_H_39_O_19_ (*m/z* 775.2070 [M+H]^+^, calcd for C_36_H_39_O_19_ 775.2080). Comparison of the ^1^H- and ^13^C-NMR spectra of **2** with those of **1** indicated that the main difference lie in the region of *β*-D-galactopyranosy moiety of **1**. The ^1^H- and ^13^C-NMR of **2** suggested a set of signals for the presence of *β*-D-glucopyranosyl group [*δ*_H_ 5.32 (1H, d, *J* = 7.5 Hz, H-1''); *δ*_C_ 66.8 (C-6''), 69.7 (C-4''), 74.1 (C-2''), 75.6 (C-5''), 76.3 (C-3''), and 101.5 (C-1'')]. In the HMBC spectrum, the long-range correlations between *δ*_H_ 5.32 (H-1'') and *δ*_C_ 133.2 (C-3); *δ*_H_ 4.53 (1H, br. s, H-1''') and *δ*_C_ 66.8 (C-6''), and *δ*_H_ 4.95 (1H, br. d, *ca*. *J* = 3 Hz, H-2''') and *δ*_C_ 164.7 (C-7'''') (Figure 2) were observed. Finally, the presences of L-rhamnopyranosyl and D-glucopyranosyl moieties in **2** were demonstrated by the acid hydrolysis like those of **1** [11,12]. Consequently, the structure of leonurusoide B was determined to be kampferol-3-*O*-(2'''-syringyl)-*α*-L-rhamnopyranosyl(1→6)-*β*-D-glucopyranoside (**2**).

**Figure 3:**
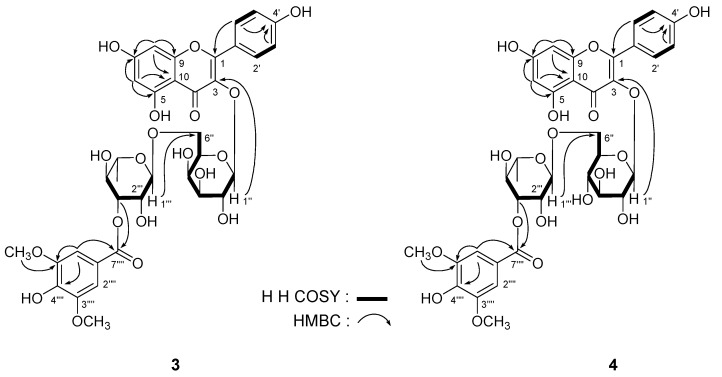
The main ^1^H-^1^H COSY and HMBC correlations of compounds **3** and **4**.

*Leonurusoides C* (**3**) and *D* (**4**) were both obtained with negative rotation [[α]^25^_D_: −27.2° (*c* = 0.38, MeOH) for **3**; and −11.0° (*c* = 0.31, MeOH) for **4**], too. The same molecular formula, C_36_H_38_O_19_, of them were determined from positive-ion HRESI-TOF-MS [*m/z* 797.1904 (**3**) and 797.1901 (**4**), both [M+Na]^+^, calcd for C_36_H_38_O_19_Na 797.1900]. Under acid hydrolysis with 1 M HCl [11,12], **3** gave L-rhamnose and D-galactose, but **4** afforded L-rhamnose and D-glucose. The ^1^H- and ^13^C-NMR (DMSO-*d*_6_, Table 2) spectra, which were assigned by various NMR experiments including ^1^H-^1^H COSY, HSQC, and HMBC spectra, indicated **3** and **4** had the same aglycon, kampferol [for **3**: 6.18 (1H, br. s, H-6), 6.42 (1H, br. s, H-8), 6.87 (2H, d, *J* = 8.0 Hz, H-3',5'), 8.06 (2H, d, *J* = 8.0 Hz, H-2',6'), 12.57 (1H, br. s, 5-OH); for **4**: 6.16 (1H, d, *J* = 1.5 Hz, H-6), 6.38 (1H, d, *J* = 1.5 Hz, H-8), 6.88 (2H, d, *J* = 8.5 Hz, H-3',5'), 7.98 (2H, d, *J* = 8.5 Hz, H-2',6'), 12.51 (1H, br. s, 5-OH)]. And both of them owned a syringyl moiety [for **3**: 3.81 (6H, s, 3'''',5''''-OC*H*_3_), 7.27 (2H, s, H-2'''',6''''); for **4**: 3.82 (6H, s, 3'''',5''''-OC*H*_3_), 7.28 (2H, s, H-2'''',6'''')]. Finally, the linkage position of *α*-L-rhamnopyranosyl, *β*-D-galacto-pyranosyl (in **3**)/*β*-D-glucopyranosyl (in **4**), and syringyl moieties were clarified on the basis of HMBC experiment (Figure 3). Consequently, the structure of leonurusoides C and D were determined to be kampferol-3-*O*-(3'''-syringyl)-*α*-L-rhamnopyranosyl(1→6)-*β*-D-galactopyranoside (**3**) and kampferol-3-*O*-(3'''-syringyl)-*α*-L-rhamnopyranosyl(1→6)-*β*-D-glucopyranoside (**4**), respectively.

**Table 2:** ^1^H and ^13^C-NMR data for compounds **3** and **4** in DMSO-*d*_6_ (500 MHz for ^1^H and 125 MHz for ^13^C).

No.	3		4	
	*δ*_C_	*δ*_H_ (*J* in Hz)	*δ*_C_	*δ*_H_ (*J* in Hz)
2	156.4	—	156.7	—
3	133.2	—	133.2	—
4	177.3	—	177.2	—
5	161.1	—	161.1	—
6	98.7	6.18 (1H, br. s)	98.7	6.16 (1H, d, 1.5)
7	164.3	—	164.3	—
8	93.7	6.42 (1H, br. s)	93.7	6.38 (1H, d, 1.5)
9	156.3	—	156.4	—
10	103.7	—	103.8	—
1'	120.7	—	120.8	—
2',6'	130.9	8.06 (2H, d, 8.0)	130.7	7.98 (2H, d, 8.5)
3',5'	115.0	6.87 (2H, d, 8.0)	115.0	6.88 (2H, d, 8.5)
4'	159.9	—	159.8	—
1''	102.0	5.34 (1H, d, 8.0)	101.5	5.32 (1H, d, 7.5)
2''	71.0	3.57 (1H, dd, 8.0, 9.0)	74.1	3.19 (1H, dd, 7.5, 8.5)
3''	72.8	3.43 (1H, dd, 3.0, 9.0)	76.3	3.24 (1H, dd, 8.5, 8.5)
4''	67.7	3.67 (1H, m, overlapped)	69.9	3.07 (1H, dd, 8.5, 9.0)
5''	73.1	3.64 (1H, m, overlapped)	75.6	3.33 (1H, m)
6''	65.0	3.29 (1H, dd, 5.0, 9.0)	67.3	3.37 (1H, dd, 5.5, 12.0)
		3.67 (1H, m, overlapped)		3.75 (1H, dd, 2.0, 12.0)
5-OH		12.57 (1H, br. s)		12.51 (1H, br. s)
1'''	99.9	4.51 (1H, br. s)	100.8	4.46 (1H, d, 1.5)
2'''	68.0	3.75 (1H, br. d, ca. 3)	67.8	3.77 (1H, dd, 1.5, 3.5)
3'''	74.7	4.84 (1H, dd, 3.0, 9.5)	74.7	4.78 (1H, dd, 3.5, 9.5)
4'''	68.9	3.49 (1H, dd, 9.5, 9.5)	68.8	3.49 (1H, dd, 8.5, 9.5)
5'''	68.3	3.58 (1H, m)	68.3	3.47 (1H, m)
6'''	17.8	1.14 (3H, d, 6.0)	17.6	1.11 (3H, d, 6.0)
1''''	119.8	—	119.9	—
2'''',6''''	107.2	7.27 (2H, s)	107.2	7.28 (2H, s)
3'''',5''''	147.3	—	147.3	—
4''''	140.4	—	140.4	—
7''''	165.3	—	165.2	—
-OCH_3_	56.0	3.81 (6H, s)	56.0	3.82 (6H, s)

**Figure 4:**
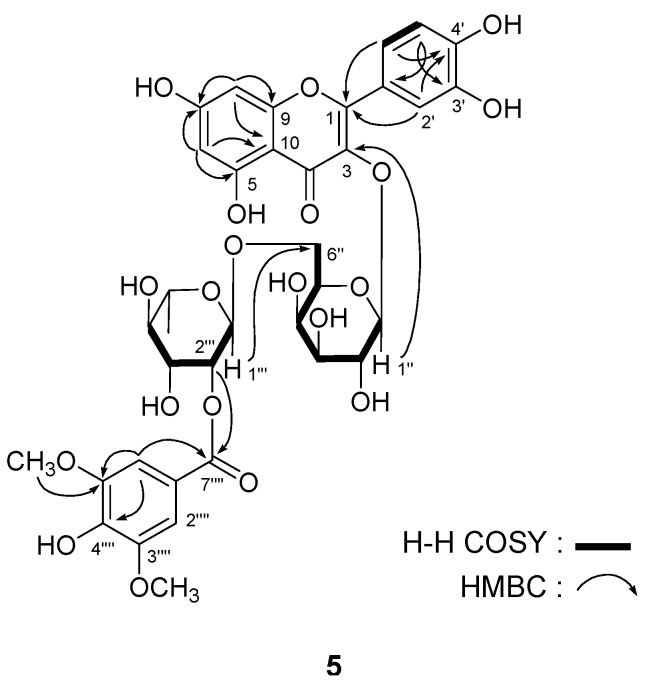
The main ^1^H-^1^H COSY and HMBC correlations of compound **5**.

*Leonurusoide E* (**5**) was obtained as a yellow powder with negative rotation [[α]^25^_D_ –18.2° (*c* = 1.09, MeOH)]. The molecular formula, C_36_H_38_O_20_, of **5** was determined by positive-ion HRESI-TOF-MS (*m/z* 813.1833 [M + Na]^+^, calcd for C_36_H_38_O_20_Na 813.1849). The ^1^H and ^13^C-NMR (DMSO-*d*_6_, Table 3), and various NMR experiments including ^1^H-^1^H COSY, HSQC, and HMBC spectra (Figure 4) of **5** revealed the presence of a syringyl group [*δ* 3.81 (6H, s, 3'''',5''''-OC*H*_3_), 7.20 (2H, s, H-2'''',6'''')], together with a hyperoside moiety {[*δ* 1.16 (3H, d, *J* = 6.0 Hz, 6'''-C*H*_3_), 4.62 (1H, d, *J* = 1.0 Hz, H-1'''], 5.35 (1H, d, *J* = 8.0 Hz, H-1''), 6.20 (1H, br. s, H-6), 6.39 (1H, br. s, H-8), 6.80 (1H, d, *J* = 8.5 Hz, H-5'), 7.54 (1H, d, *J* = 2.0 Hz, H-2'), 7.63 (1H, dd, *J* = 2.0, 8.5 Hz, H-6), 12.58 (1H, br. s, 5-OH)]}.

**Table 3:** ^1^H and ^13^C-NMR data for compound **5** in DMSO-*d*_6_ (500 MHz for ^1^H and 125 MHz for ^13^C).

	*δ*_C_	*δ*_H_ (*J* in Hz)		*δ*_C_	*δ*_H_ (*J* in Hz)
2	156.2	—	3''	72.9	3.43 (1H, dd, 3.0, 9.5)
3	133.4	—	4''	67.8	3.68 (1H, m, overlapped)
4	177.2	—	5''	73.2	3.64 (1H, m, overlapped)
5	161.1	—	6''	65.1	3.33 (1H, dd, 5.5, 9.5)
6	98.6	6.20 (1H, br. s)			3.67 (1H, m, overlapped)
7	164.3	—	1'''	96.8	4.62 (1H, d, 1.0)
8	93.5	6.39 (1H, br. s)	2'''	72.6	4.93 (1H, dd, 1.0, 3.0)
9	156.2	—	3'''	68.6	3.65 (1H, m, overlapped)
10	103.7	—	4'''	72.4	3.28 (1H, dd, 9.0, 9.5)
1'	121.0	—	5'''	68.4	3.54 (1H, m)
2'	115.9	7.54 (1H, d, 2.0)	6'''	18.0	1.16 (3H, d, 6.0)
3'	144.7	—	1''''	119.2	—
4'	148.4	—	2'''',6''''	107.0	7.20 (2H, s)
5'	115.0	6.80 (1H, d, 8.5)	3'''', 5''''	147.4	—
6'	121.7	7.63 (1H, d, 2.0, 8.5)	4''''	140.7	—
5-OH		12.58 (1H, br. s)	7''''	164.7	
1''	101.9	5.35 (1H, d, 8.0)	-OCH_3_	55.9	3.81 (6H, s)
2''	71.0	3.60 (1H, dd, 8.0, 9.0)			

The position of syringyl group was determined by the HMBC experiment, which showed long-range correlations between the protons of position-2'" [*δ*_H_ 4.93 (1H, dd, *J* = 1.0, 3.0 Hz)] and ester carboxyl carbon [*δ*_C_ 164.7 (C-7"'')]. Finally, acid hydrolysis of **5** with 1 M HCl afforded L-rhamnose and D-galactose determined by HPLC analysis [11,12]. Consequently, the structure of leonurusoide E was determined to be quercetin-3-*O*-(2'''-syringyl)-*α*-L-rhamnopyranosyl(1→6)-*β*-D-galactopyranoside (**5**). The known acylated flavonoid glycoside compound **6** was identified as 2'''-syringylrutin by comparing its physical properties and spectral data with that reported in the literature [6].

Intracellular excess lipid accumulation (especially in liver and muscle) is a mediator of metabolic syndrome, which comprises a cluster of risk factors such as diabetes, hyperlipidemia, and hypertension. Free fatty acid induced TG accumulation HepG2 cells is commonly used for research on lipid metabolism regulation effects. The TG accumulation inhibitory effects of the isolates **1**–**6** were tested. All compounds showed inhibitory effects on TG accumulation in free fatty acid induced HepG2 cells (Table 4).

**Table 4:** TG accumulation inhibitory effects of **1**–**6** in HepG2 cells.

	Concentration ( *μ*M)	Inhibitory rate (%)
Control	0	0.0 ± 1.2
Orlistat	1	43.3 ± 4.1 *
1	10	61.4 ± 10.0 **
2	10	52.8 ± 4.2 *
3	10	56.9 ± 9.6 **
4	10	51.7 ± 6.9 *
5	10	49.1 ± 4.9 *
6	10	44.3 ± 4.3 *

Intracellular TG accumulation inhibitory rate of control group was set as 0%. Values represent the mean ± SD of six determinations. * *p* < 0.05; ** *p* < 0.01 *vs.* control group.

## 3. Experimental

### 3.1. General

Optical rotations were measured on a Rudolph Autopol^®^ IV automatic polarimeter. IR spectra were recorded on a Varian 640-IR FT-IR spectrophotometer. UV spectra were obtained on a Varian Cary 50 UV-Vis spectrophotometer. NMR spectra were determined on a Bruker 500 MHz NMR spectrometer at 500 MHz for ^1^H and 125 MHz for ^13^C-NMR, with TMS as an internal standard. Positive- and Negative-ion HRESI-TOF-MS were recorded on an Agilent Technologies 6520 Accurate-Mass Q-Tof LC/MS spectrometer. Column chromatographies (CCs) were performed on macroporous resin D101 (Haiguang Chemical Co., Ltd., Tianjin, China), silica gel (48–75 μm, Qingdao Haiyang Chemical Co., Ltd., Qingdao, China), Sephadex LH-20 (Ge Healthcare Bio-Sciences, Uppsala, Sweden), and ODS (40-63 μm, YMC Co., Ltd., Tokyo, Japan). A Cosmosil 5C18-MS-II (20 mm i.d. × 250 mm, Nakalai Tesque, Inc., Tokyo, Japan) preparative HPLC column was used to purify the constituents. Pre-coated TLC plates with Silica gel GF_254_ (Tianjin Silida Technology Co., Ltd., Tianjin, China) were used to detect the purity of isolate; visualization was achieved by spraying with 10% aqueous H_2_SO_4_-EtOH, following by heating.

### 3.2. Plant Material

The aerial parts of *L. japonicus* Houtt were purchased from Anguo Medicinal Market, Hebei Province, China and identified by Dr. Li Tianxiang. The voucher specimen was deposited at the Academy of Traditional Chinese Medicine of Tianjin University of TCM (No. 20110909).

### 3.3. Extraction and Isolation

The aerial parts of *L. japonicus* (6.0 kg) were finely cut and refluxed with 50% ethanol-water (48 L, 2 h) three times. Evaporation of the solvent under reduced pressure provided a 50% ethanol-water extract (600.0 g). The 50% ethanol-water extract (480.1 g) was partitioned into a CHCl_3_-H_2_O (1:1, v/v) mixture to furnish a CHCl_3_-soluble fraction (46.1 g) and an aqueous phase (430.2 g). The aqueous soluble fraction (362.0 g) was subjected to D101 resin CC, and eluted with H_2_O, 70% and 95% EtOH, successively, and the H_2_O (255.1 g), 70% EtOH (78.3 g) and 95% EtOH (32.2 g) eluted fractions were obtained, respectively. The 70% EtOH eluted fraction (75.0 g) was separated by silica gel CC [CHCl_3_ → CHCl_3_-MeOH (100:5, v/v) → CHCl_3_-MeOH-H_2_O (10:3:1 → 7:3:1 → 6:4:1, v/v/v, the lower layer) → MeOH] to afford nine fractions (Fr. 1−9). Fraction 2 (15.0 g) was separated by ODS CC [MeOH-H_2_O (10% → 20% → 30% → 40% → 50% → 60% → 100%, v/v)], and 12 fractions (Fr. 2-1−2-12) were obtained. Fraction 2-9 (0.97 g) was separated by preparative HPLC (PHPLC) [CH_3_CN-H_2_O (22:78, v/v)] to afford eight fractions (Fr. 2-9-1−2-9-8). Fractions 2-9-7 (85.3 mg) and 2-9-8 (90.1 mg) were further purified by PHPLC [MeOH-H_2_O (54:46, v/v)] to give leonurusoides E (**5**, 24.2 mg) and C (**3**, 9.7 mg), respectively. Fraction 2-10 (1.0 g) was separated by PHPLC [CH_3_CN-H_2_O (23:77, v/v)] to yield 10 fractions [Fr. 2-10.1−2-10.10]. Fraction 2-10-4 (440.1 mg) was purified by PHPLC [MeOH-H_2_O (46:54, v/v)] to give leonurusoide E (**5**, 34.1 mg). Fraction 2-10-10 (61.8 mg) was further separated by PHPLC [MeOH-H_2_O (47:53, v/v) to afford leonurusoide D (**4**, 6.7 mg). Fraction 2-11 (1.13 g) was separated by PHPLC [CH_3_CN-H_2_O (23:77, v/v)] to give six fractions (Fr. 2-11-1−2-11-6). Fractions 2-11-1 (381.8 mg) and 2-11-3 (56.2 mg) were purified by PHPLC [MeOH-H_2_O (52:48, v/v)] to give 2'''-syringylrutin (**6**, 5.8 mg) and leonurusoide A (**1**, 13.4 mg). Fraction 2-11-4 (188.9 mg) was further subjected to PHPLC [MeOH-H_2_O (53:47, v/v)] to afford leonurusoide B (**2**, 3.8 mg).

*Leonurusoide A* (**1**). Yellow powder. [α]^25^_D_ –13.0° (*c* = 0.54, MeOH); IR ν_max_ (KBr) cm^–1^: 3356, 2922, 2840, 1699, 1654, 1608, 1516, 1457, 1361, 1281, 1210, 1180, 1116, 1056, 841, 762; UV λ_max_ (MeOH) nm (log *ε*): 351 (4.00), 268 (4.24). ^1^H-NMR (DMSO-*d*_6_) and ^13^C-NMR (DMSO-*d*_6_) spectroscopic data, see Table 1. HRESI-TOF-MS: Positive-ion mode *m/z* 775.2076 [M+H]^+^ (calcd for C_36_H_39_O_19_ 775.2080).

*Leonurusoide B* (**2**). Yellow powder. [α]^25^_D_ –16.6° (*c* = 0.21, MeOH); IR ν_max_ (KBr) cm^–1^: 3218, 2948, 2841, 1699, 1653, 1608, 1569, 1517, 1458, 1361, 1281, 1210, 1180, 1113, 1055, 841, 762; UV λ_max_ (MeOH) nm (log *ε*): 349 (3.88), 267 (4.13). ^1^H-NMR (DMSO-*d*_6_) and ^13^C-NMR (DMSO-*d*_6_) spectroscopic data, see Table 1. HRESI-TOF-MS: Positive-ion mode *m/z* 775.2070 [M+H]^+^ (calcd for C_36_H_39_O_19_ 775.2080).

*Leonurusoide C* (**3**). Yellow powder. [*α*]_D_^25^ –27.2° (*c* = 0.38, MeOH); IR ν_max_ (KBr) cm^–1^: 3231, 2972, 2860, 1699, 1653, 1608, 1588, 1516, 1457, 1362, 1281, 1210, 1181, 1116, 1056, 765, 680; UV λ_max_ (MeOH) nm (log *ε*): 348 (4.16), 268 (4.41). ^1^H-NMR (DMSO-*d*_6_) and ^13^C-NMR (DMSO-*d*_6_) spectroscopic data, see Table 2. HRESI-TOF-MS: Positive-ion mode *m/z* 797.1904 [M+Na]^+^ (calcd for C_36_H_38_O_19_Na 797.1900).

*Leonurusoide D* (**4**). Yellow powder. [α]^25^_D_ –11.0° (*c* = 0.31, MeOH); IR ν_max_ (KBr) cm^–1^: 3322, 2921, 2843, 1699, 1653, 1589, 1518, 1458, 1357, 1290, 1213, 1181, 1118, 1055, 772, 677; UV λ_max_ (MeOH) nm (log *ε*): 346 (3.86), 267 (4.13). ^1^H-NMR (DMSO-*d*_6_) and ^13^C-NMR (DMSO-*d*_6_) spectroscopic data, see Table 2. HRESI-TOF-MS: Positive-ion mode *m/z* 797.1901 [M+Na]^+^ (calcd for C_36_H_38_O_19_Na 797.1900).

*Leonurusoide E* (**5**). Yellow powder. [α]^25^_D_ –18.2° (*c* = 1.09, MeOH); IR ν_max_ (KBr) cm^–1^: 3323, 2960, 1697, 1652, 1608, 1516, 1457, 1424, 1340, 1302, 1206, 1117, 1070, 815, 764, 675; UV λ_max_ (MeOH) nm (log *ε*): 361 (4.24), 265 (4.43). ^1^H-NMR (DMSO-*d*_6_) and ^13^C-NMR (DMSO-*d*_6_) spectroscopic data, see Table 3. HRESI-TOF-MS: Positive-ion mode *m/z* 813.1833 [M+Na]^+^ (calcd for C_36_H_38_O_20_Na 813.1849).

#### Acid Hydrolysis of **1**–**5**

A solution of leonurusoides A–E (**1**–**5**, each 1.5 mg) in 1 M HCl (1 mL) was heated under reflux for 3 h, respectively. The reaction mixture was neutralized with Amberlite IRA-400 (OH^–^ form) and removed by filtration. The aqueous layer was subjected to the HPLC analysis under the following condition, respectively: HPLC column, Kaseisorb LC NH_2_-60-5 (4.6 mm i.d. × 250 mm, Tokyo Kasei Co. Ltd., Tokyo, Japan); detection, optical rotation [Chiralyser (IBZ Messtechnik GMBH, Mozartstrasse 14-16 D-30173 Hannover, Germany)]; mobile phase, CH_3_CN-H_2_O (75:25, v/v); flow rate 1.0 mL/min. Identification of L-rhamnose (i) and D-galactose (iii) from **1**, **3** and **5**; L-rhamnose (i) and D-glusose (ii) from **2** and **4** presented in the aqueous was carried out by comparison of its retention time and optical rotation with that of authentic samples, *t_R_*: (i) 7.5 min (negative, L-rhamnose), (ii) 13.9 min (positive, D-glusose), and (iii) 14.5 min (positive, D-galactose).

### 3.4. TG Accumulation Inhibitory Effects Assay

The hepatic cell line HepG2 (IBMS, CAMS/PUMC, Beijing, China) were maintained in high glucose Minimum Essential Medium (MEM) supplemented with 10% fetal bovine serum (FBS) and 1% penicillin-streptomycin under a humidified atmosphere of 5% CO_2_ in air. After growth to 80% confluence, cells were seeded at 4 × 10^4^ cells/mL on 48-well dish. After 24 h incubation, the medium was switched to high glucose MEM and supplemented with 10% FBS and 0.2 mM oleic acid sodium salt, together with sample DMSO solution (final concentration of DMSO was less than 0.1%). After 48 h incubation, the amount of intracellular triglycerides was determined with a Triglycerides kit (BioSino Bio-technology and Science Inc., Beijing, China) after cell lysis.

#### Statistical Analysis

Values are expressed as mean ± S.D. All the grouped data were statistically performed with SPSS 11.0. Significant differences between means were evaluated by one-way analysis of variance (ANOVA) and Tukey’s Studentized range test was used for post hoc evaluations. *p* < 0.05 was considered to indicate statistical significance.

## 4. Conclusions

In summary, five new syringyl acylated flavonoid glycosides **1**−**5**, together with the known compound, 2'''-syringylrutin (**6**) were obtained from the aerial parts of *L. japonicas*. Although various acylated flavonol glycosides distribute widely in the plant kingdom [13], syringates such as the isolates **1**−**6** are quite rare. Compound **6** and quercetin 3-(3'''-syringylrobinobioside), with a syringyl acylated at positions 2 and 3 of rhamnose, respectively, are the first examples of natural flavonol glycosides from the same genus, *Leonurus* [6,7], and are only found in the genus. All of the syringyl acylated flavonoid glycosides **1**−**6** showed TG accumulation inhibitory effects in free fatty acid-induced HepG2 cells.
